# The role of endothelin-1 in hyperoxia-induced lung injury in mice

**DOI:** 10.1186/1465-9921-7-45

**Published:** 2006-03-27

**Authors:** Walid Habre, Ferenc Peták, Isabelle Ruchonnet-Metrailler, Yves Donati, Jean-Francois Tolsa, Eniko Lele, Gergely Albu, Morice Beghetti, Constance Barazzone-Argiroffo

**Affiliations:** 1 Pediatric Anesthesia Unit, Geneva Children's Hospital, University Hospitals of Geneva, 6, Rue Willy Donze, CH-1205, Geneva, Switzerland; 2 Department of Medical Informatics and Engineering, University of Szeged, Koranyi fasor 9, H-6720, Szeged, Hungary; 3 Department of Immunology and Pathology, University of Geneva, 1 rue Michel-Servet, CH-1211, Geneva 14, Switzerland; 4 Department of Pediatrics, University Hospital of Lausanne, Rue du Bugnon 46, 1011 Lausanne, Switzerland; 5 Pediatric Cardiology Unit, Department of Pediatrics, Geneva Children's Hospital, 6, Rue Willy Donze, CH-1205, Geneva, Switzerland; 6 Pediatric Pulmonology Unit, Department of Pediatrics, Geneva Children's Hospital, 6, Rue Willy Donze, CH-1205, Geneva, Switzerland

## Abstract

**Background:**

As prolonged hyperoxia induces extensive lung tissue damage, we set out to investigate the involvement of endothelin-1 (ET-1) receptors in these adverse changes.

**Methods:**

Experiments were performed on four groups of mice: control animals kept in room air and a group of mice exposed to hyperoxia for 60 h were not subjected to ET-1 receptor blockade, whereas the dual ETA/ETB-receptor blocker tezosantan (TEZ) was administered via an intraperitoneal pump (10 mg/kg/day for 6 days) to other groups of normal and hyperoxic mice. The respiratory system impedance (Zrs) was measured by means of forced oscillations in the anesthetized, paralyzed and mechanically ventilated mice before and after the iv injection of ET-1 (2 μg). Changes in the airway resistance (Raw) and in the tissue damping (G) and elastance (H) of a constant-phase tissue compartment were identified from Zrs by model fitting.

**Results:**

The plasma ET-1 level increased in the mice exposed to hyperoxia (3.3 ± 1.6 pg/ml) relative to those exposed to room air (1.6 ± 0.3 pg/ml, p < 0.05). TEZ administration prevented the hyperoxia-induced increases in G (13.1 ± 1.7 vs. 9.6 ± 0.3 cmH_2_O/l, p < 0.05) and H (59 ± 9 vs. 41 ± 5 cmH_2_O/l, p < 0.05) and inhibited the lung responses to ET-1. Hyperoxia decreased the reactivity of the airways to ET-1, whereas it elevated the reactivity of the tissues.

**Conclusion:**

These findings substantiate the involvement of the ET-1 receptors in the physiopathogenesis of hyperoxia-induced lung damage. Dual ET-1 receptor antagonism may well be of value in the prevention of hyperoxia-induced parenchymal damage.

## Background

In many lung diseases, clinicians are faced with the need to administer high inspired fractions of oxygen to overcome hypoxemia, although the breathing of oxygen-enriched air is known to induce alveolar damage and airway irritation in animals and humans [1]. Hyperoxia-induced lung injury is mediated by numerous mediators, leading to alveolar cell death, alveolo-capillary disruption and a lung function deterioration [2]. The level of endothelin-1 (ET-1), a major mediator regulating both the vascular [3,4] and the airway smooth muscle tone [5-7], has been reported to be elevated following the exposure of endothelial cells to hyperoxia [8], and also in different *in vivo *experimental models of acute lung injury [9,10]. To date, the role of ET-1 has been primarily documented in cardiovascular diseases, and particularly in pulmonary hypertension. The discovery of ET-1 receptor blocking agents is now recognized as a milestone in the treatment of this latter disease.

Besides being a major mediator regulating the vascular tone, ET-1 also plays a major role in the regulation of the airway caliber [6,7,11-14]. Accordingly, the use of ET-1 receptor blockers may also be of promise for the prevention of the deleterious effects of ET-1 on the lungs; in particular, the effects of the exogenous administration of ET-1 on the lung function have been successfully inhibited by the administration of these agents [11,14]. However, the preventive potential of ET-1 receptor blockers in combating elevated levels of endogenous ET-1 in the presence of lung diseases such as hyperoxia-induced lung injury has not been characterized.

In a murine model of hyperoxia-induced lung injury, we earlier demonstrated that features of apoptosis and necrosis affect alveolar cells [15]. These histopathological changes are reflected in deteriorated parenchymal mechanical properties, whereas the airway function is preserved [16]. Since ET-1 has been shown to be associated with the apoptosis of endothelial cells [17] and, similarly to hyperoxia, it also induces a parenchymal function impairment [7,11,18], the involvement of this peptide in the pathogenesis of hyperoxia-induced lung injury may be anticipated. Thus, we hypothesized that ET-1 is involved in the lung function deterioration observed following the exposure of mice to hyperoxia. To test this hypothesis, we compared naive animals exposed to hyperoxia with those where to which a dual ETA/ETB receptor blocker, tezosentan (TEZ), had been administered. Moreover, to assess whether hyperoxia affects the lung responsiveness, we also set out to characterize the changes in the lung mechanical response to exogenous ET-1.

## Methods

### Animal preparations

The measurements were performed on 6- to 8-wk-old female C57BL/6 mice mice weighing 20–23 g. The protocol had been approved by the institutional ethics committee for animal experiments and by the veterinary office of the Canton of Geneva. Mice were randomly assigned to one or other of the following four protocol groups. There was no statistically significant difference in body weight between the animals enrolled in the different protocol groups. Two groups of animals kept in room air (group C, n = 8) or were exposed to hyperoxia for 60 h (group Hox, n = 10) were not subjected to ET-1 receptor blockade, dual ETA/ETB-receptor blocker TEZ was administered continuously for 6 days via an intraperitoneal pump (10 mg/kg/day) to two other groups of mice, likewise kept in room air (group CT, n = 6) or exposed to hyperoxia for 60 h (group HoxT, n = 7). In these latter two groups of mice, an osmotic intraperitoneal pump implanted under sterile surgery was used to deliver TEZ at a rate of 1 μl/h from a 10 mg/ml solution in phosphate-buffered saline. The solvent alone was administered in an identical manner to the animals in the groups C and Hox by performing the surgical implantation of an identical intraperitoneal pump as in the groups receiving TEZ. Our previous investigations led us to expose the mice to 100% oxygen for 60 h in order to induce marked lung tissue damage without lethal consequences [16].

Hyperoxia was induced by exposure of the animals to 100% oxygen in a sealed (8-liter) Plexiglas chamber under minimal oxygen inflow and outflow (0.5 l/min). The CO_2 _level in the box was maintained at 1% by using a CO_2 _absorber (Drägersorb 800, Dräger Medizintechnik, Lübeck, Germany). Food and water were available *ad libitum*. On the day of the experiment, the mice were anesthetized with an intraperitoneal injection of pentobarbital sodium (50 mg/kg). Tracheostomy was performed, and a polyethylene cannula (30 mm long, 1.17 mm ID) was inserted into the trachea. The mice were then mechanically ventilated (model 683, Harvard Apparatus, South Natick, MA) with a tidal volume of 10 μl/g at a frequency of 180/min. Muscle paralysis was accomplished through the intraperitoneal administration of pancuronium bromide (1 mg/kg). To avoid acute hypoxia in the hyperoxic mice, the animals were kept in 100% oxygen during the surgical preparation and mechanical ventilation. A femoral vein was cannulated with a 26-gauge catheter for the iv delivery of ET-1.

### Measurement of ET-1 levels in blood

Venous blood samples were collected retroorbitally (200 μl of blood) on EDTA and centrifuged (900 g at room temperature for 10 min). The separated plasma was stored at -20°C until assay. The ET-1 level in the plasma was then determined by an Elisa technique (Human Endothelin Immunoassay, Catalog number QET00B, R&D Systems Inc. Minneapolis MN). Reference values were established from blood samples taken from another group of mouse identical to those involved in the present study but receiving no anesthesia or treatment at all.

### Forced oscillatory measurements

The forced oscillatory setup for the measurement of respiratory mechanical impedance (Zrs) was described in detail previously [16,19]. Briefly, a three-way tap (Becton-Dickinson, model 394600, Helsinborg, Sweden) was used to switch the tracheal cannula from the respirator to a loudspeaker-in-box system at end-expiration. A 28-cm-diameter loudspeaker (250 W) enclosed in a plastic box served as the pressure generator. The loudspeaker generated a small-amplitude pseudorandom signal (25 integer-multiple frequency components between 1 and 25 Hz) through a 100-cm-long, 1.17-mm-ID polyethylene tube (Becton-Dickinson, model 6253, Rutherford, NJ). Two identical pressure transducers (model 33NA002D, ICSensors, Milpitas, CA) were used to measure the lateral pressures at the loudspeaker (P_1_) and at the tracheal end (P_2_) of the wave tube. The P_1 _and P2 signals were low-pass filtered (5th-order Butterworth, 25-Hz corner frequency) and sampled with a custom made analog-digital board (with core components from Analog Devices Inc., Norwood, MA, USA) of a computer at a rate of 256 Hz. Fast Fourier transformation with 1-s time windows and 90% overlapping was used to compute the pressure transfer functions (P_1_/P_2_) from the 3-s recordings. The P_1_/P_2 _spectra were used to calculate Zrs as the load impedance of the wave tube [16,19]. Four to six Zrs values were collected in each mouse for averaging. To avoid possible bias in the impedance calculation due to the accumulated oxygen in the wave tube, the oxygen administration was suspended and the lungs were washed with room air for 30 s before each recording. Intervals of at least 2 min were interposed between each two Zrs measurements.

The airway and tissue properties were quantified by fitting a model to the Zrs spectra under each experimental condition [11,16,18-20]. The model contained airway resistance (Raw) and inertance (Iaw) in series with the tissue damping (G) and elastance (H) of a constant-phase tissue model [20]. Tissue hyteresivity (η) was calculated as η = G/H [21]. Impedance data at frequencies coinciding with the heart rate and its harmonics were omitted from the fitting if the cardiac activity caused a low signal-to-noise ratio at these frequencies and thus, leading to elevations in the scatter of the Zrs data at these points. The contribution of the measurement apparatus, including the tracheal cannula to the reported Raw values, was 0.118 cmH_2_O.s/ml.

### Study protocol

After surgery, a 10–15-min period was allowed for the mice to reach a steady-state condition. A deep lung inflation was then performed by superimposing two inpiratory cycles to standardize volume history. Four Zrs recordings were then made to establish the baseline. ET-1 at a dose of 2 μg dissolved in 0.1 ml saline was next administered via the femoral vein to induce lung constriction. Following ET-1 injection, Zrs was measured at 30 s, and then at 1-min intervals until 6 min, and at 2 min intervals until 20 min. The Zrs data collected under baseline conditions were averaged and used for the model fitting. The individual Zrs data were fitted with the model after the administration of the ET-1 challenge.

### Statistical analysis

Scatters in the parameters are expressed in SE values. The plasma ET-1 levels were compared by using the Student t-test. Two-way repeated measures analysis of variance (ANOVA) was used, with the administration of TEZ and ET-1 as the first and second variables, respectively, to establish the effects of TEZ pretreatment on the respiratory mechanical parameters under baseline conditions and following the ET-1 challenge. The Student-Newman-Keuls multiple comparison procedure was employed to compare the lung mechanical parameters under different conditions. In each test, a significance level of p < 0.05 was applied.

## Results

The plasma ET-1 level was increased significantly (p < 0.05) in the mice exposed to hyperoxia (3.3 ± 1.6 pg/ml, n = 11) compared with those kept in room air (1.6 ± 0.3 pg/ml, n = 8).

**Figure 1:**
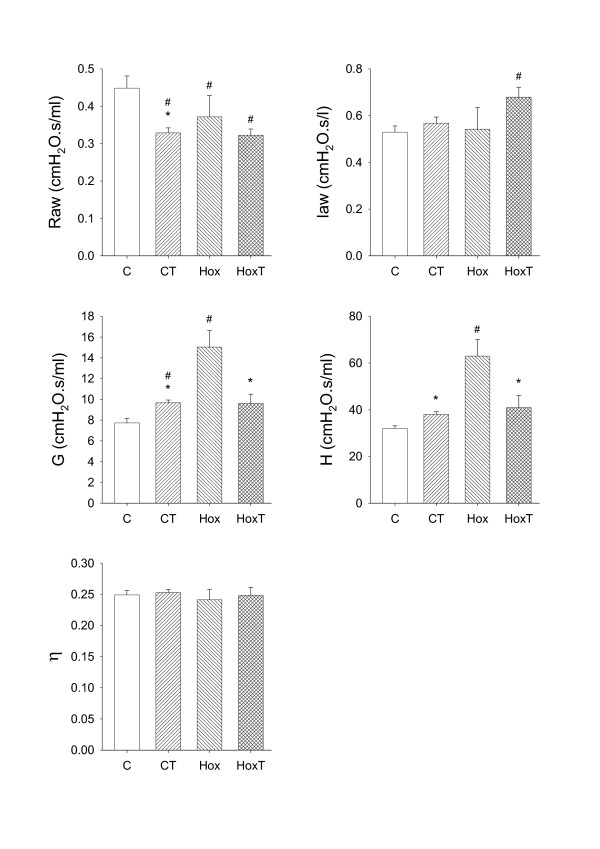
The baseline values of the airway and tissue mechanical parameters obtained in control mice without pretreatment (C), in control mice pretreated with the endothelin receptor blocker tezosentan (CT), and in mice exposed to hyperoxia without pretreatment (Hox) or with pretreatment with tezosentan (HoxT). Raw: airway resistance, Iaw: airway inertance, G: tissue damping, H: tissue elastance, η: tissue hyteresivity. *: p < 0.05 for the effect tezosentan treatment, #: p < 0.05 vs the parameter value obtained in group C.

The airway and tissue mechanical parameters obtained under the control conditions in the four protocol groups are demonstrated in Fig. 1. In the mice kept in room air, TEZ decreased Raw and increased G and H significantly (p < 0.05). In agreement with our previous findings [16], hyperoxia induced mild, but statistically significant decreases in Raw (p < 0.05), whereas the tissue resistive and elastic parameters increased markedly. The hyperoxia-induced increases in G and H were significantly inhibited by TEZ administration. η was not affected by hyperoxia or TEZ.

**Figure 2:**
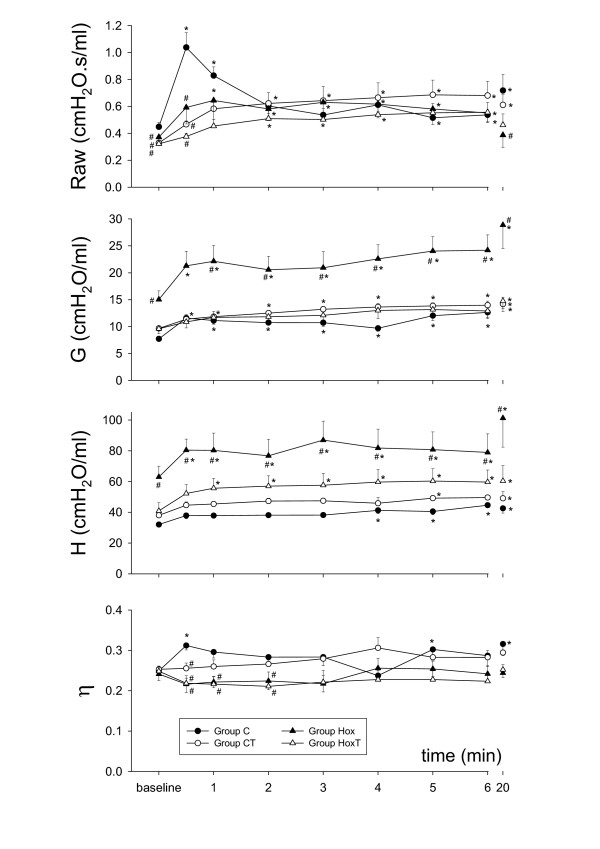
Temporal changes in the airway and tissue mechanical parameters after iv endothelin-1 administration. Raw: airway resistance, G: tissue damping, H: tissue elastance, η: tissue hyteresivity. *: p < 0.05 vs the baseline value within a group; #: p < 0.05 vs group C (control, no tezosentan).

The ET-1-induced changes in the airway and tissue mechanical parameters are depicted in Fig. 2. The ET-1-induced responses in Raw were greatly influenced both by the presence of hyperoxia and by TEZ. In the control group of mice (group C), the administration of ET-1 induced an immediate increase in Raw (129 ± 11% at 30 s), with a subsequent rapid return to the control level (at 2 min). The administration of ET-1 led to significantly smaller increases in Raw in the other three groups of mice (43 ± 7%, 56 ± 24 and 17 ± 6% in groups Hox, CT and HoxT at 30 s, respectively), the altered temporal profile being reflected in a slightly delayed and more sustained response. As regards the tissue parameters G and H, the hyperoxia-induced elevation in their baseline values in group Hox were associated with further marked elevations in response to ET-1 (41 ± 10% and 33 ± 8% for G and H at 30 s, respectively), which were maintained until the end of the study period. TEZ administration not only prevented the elevations in the baseline G and H values, but also blunted their elevations in response to ET-1, with a temporal profile similar to that observed in the group C. Following ET-1 administrations η exhibited significant increases only in the animals of group C at 30 s, while this parameter showed no statistically significant changes in the other groups of mice.

## Discussion

The involvement of ET-1 in the hyperoxia-induced lung function deterioration was characterized in detail in the present study by assessing the changes in the airway and tissue mechanics separately following exposure to oxygen and in response to an exogenous ET-1 challenge in naïve animals and in mice pretreated with an ET-1 receptor blocker. Partitioning of the airway and tissue responses revealed highly dissociated effects of hyperoxia on these compartments: in the absence of ET-1 receptor blockade, the hyperoxia-induced mild bronchodilations were associated with significantly compromised parenchymal mechanics. Continuous administration of an ET-1 receptor blocker prevented the deterioration induced by hyperoxia in the parenchymal mechanics, while it had no effect on the airways. Assessment of the airway and tissue responses following an iv ET-1 challenge also revealed the contradictory behavior of these compartments: while the airways displayed a diminished reactivity for this constrictor stimulus during hyperoxia, the lung parenchyma exhibited a significantly enhanced constrictor response.

The Zrs curves and the extracted airway and the tissue parameters are in excellent agreement with the results of our earlier report [16], and are consistent with those obtained by other laboratories in this species under baseline conditions [22-25]. Furthermore, the hyperoxia-induced changes in the airway and tissue parameters in naïve mice are similar to those reported by our group previously [16], confirming the need for the separate measurement of the airway and tissue mechanical parameters in order to describe the lung mechanical impairment precisely under these conditions. Previous studies validating the reliability of the separation of airway and parenchymal parameters via model-based evaluation of the low-frequency input impedance spectra revealed that Raw accurately characterizes the overall airway resistance [26]. Nevertheless, it has also been demonstrated that the tissue parameters of a single-compartment constant-phase model may be affected by severe peripheral airway heterogeneities, which may develop in the presence of acute lung injury [27]. Since heterogeneities are reflected primarily in the elevated η values, and we observed no difference in this parameter between the protocol groups under the baseline conditions (Fig. 1), it can be concluded that hyperoxia induced rather uniform lung damage with no alterations in the ratio of the energy dissipation to energy storage in the respiratory tissues. The significant η elevation observed in Group C 30 seconds after ET-1 injection suggests that marked ventilation heterogeneities may have contributed to the marked increases in G obtained in this group at early stage of the challenge.

It has been now established that, besides its vasoconstrictive potential, ET-1 compromises the lung mechanics [7,11,14,18] and it may therefore play a role in the regulation of the airway tone. Separate assessment of the airway and tissue responses to ET-1 in mice previously has only been achieved by Nagase et al. with the use of alveolar capsules [7]. In contrast with our findings that ET-1 induced primarily an increase in Raw, with a smaller increase in G and no change in H in the control mice, Nagase et al. observed similar increases in the airway and parenchymal mechanical parameters at all ET-1 doses administered iv to naive animals. Besides the substantial difference in lung configuration due to the chest opening [28], further methodological differences between the studies may explain this discrepancy. Nagase et al. estimated the airway and tissue responses to ET-1 by measuring local alveolar pressures. Nevertheless, the gluing of alveolar capsules with a diameter comparable to the sternocostal area of the lungs will not only certainly influences the reliability of airway-tissue separation, but also probably biases the estimation of the overall lung mechanics. Further, sampling of one or two alveolar regions, as performed previously [7], in inhomogenously constricted lungs [19] makes the capsule-based partitioning highly incidental [29].

The literature data regarding the effects of hyperoxia on the responsiveness of the airways to exogenous constrictor stimuli are somewhat conflicting. Oxygen exposure has been reported to have no effect on the bronchial reactivity [30-32], to enhance the responsiveness of the airways [33-36] and even to reduce the contractile responses of the bronchi [37] to exogenous constrictor agonists stimulating the muscarinic receptors. Many factors may contribute to this situation. The degree of maturity of the experimental animals has been suggested to influence the airway reactivity, with immature rodents being more hyperreactive than adults [31,35-37]. The involvement of different mediators released by the inflammatory cells following hyperoxia may also have an impact on the lung responses to exogenous stimuli [31]; these effects are found to be diminished in *in vitro *studies [31,33]. The results of the present study indicate that the methodological differences between the techniques used to assess the airway reactivity are the most likely explanation of this controversy.

**Figure 3:**
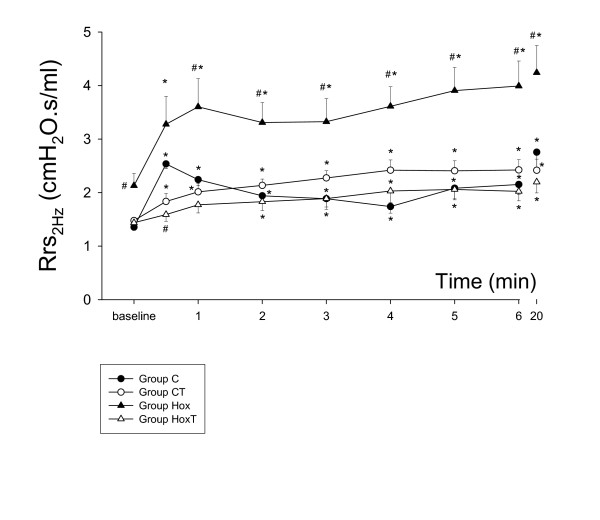
Temporal changes in the total respiratory system resistance at 2 Hz after iv endothelin-1 administration. *: p < 0.05 vs. the baseline value within a group; #: p < 0.05 vs group C (control, no tezosentan).

All of the *in vivo *previous studies describing changes in the bronchial reactivity in various experimental models of hyperoxia measured overall parameters, such as the total respiratory [34-36] or pulmonary system resistance [30,32], to characterize the mechanical changes in the lungs. These mechanical parameters incorporate the flow resistance of the airways and the dissipative properties of the tissues [7,11,15,16,18-20,28,29]. Hence, dissociated changes in these components cannot be identified, since any change in tissue dissipation mask those in the airway properties. Accordingly, opposite changes in these components blunt those in the overall parameters, or they may even be totally extinguished if they are opposite and equal in magnitude. Our results demonstrate the presence of such opposite changes in the responsiveness of the airway and tissue compartments in the presence of oxygen toxicity. Indeed, expression of the hyperoxia-induced changes in the ET-1-responsiveness via calculation of the total respiratory system resistances at the breathing frequency (Rrs = Raw + G/ωα, where ω = 4π at 2 Hz and α = 2/π arctan [H/G]) in Fig. 3 may lead to misinterpretation of the lung responses to contrictor stimuli. In fact, the apparent hyperreactivity of the lung is attributed to the presence of the enhanced responsiveness of the lung parenchyma and not to the elevated reactivity of the airways. As far as we are aware, this is the first *in vivo *study that has established the changes in the airway and tissue mechanics separately following a constrictor challenge during hyperoxia. The fact of the opposite changes in the altered reactivity of the airways and the respiratory tissues emphasizes the need for a separate assessment of the mechanical responses in these compartments, and also indicates that previous results should be interpreted with some caution.

The underlying mechanism responsible for the opposite changes in the airway and the tissue reactivity to ET-1 following hyperoxia is not completely clear. ET-1 generates its effect by stimulating both ETA and ETB receptor subtypes. The antagonism of the ETA receptors inhibits the ET-1-induced contraction in parenchymal strips, whereas this treatment is ineffective in isolated bronchi [38], indicating that the antagonist ETA receptors are expressed in greater density in the lung parenchyma, while the ETB receptors are expressed more markedly in the airways. Indeed, autoradiographic studies have revealed the predominance of the ETB receptors in the trachea in mice [39]. The different distributions of the receptor subtypes is of importance, since lung inflammation has been demonstrated to decrease the density of the ETB receptors [39] and to increase that of the ETA receptors [40]. Since hyperoxia also leads to lung inflammation, it is possible that the diminished airway responses are due to the decreased density of the ETB receptors, whereas the enhanced parenchymal responsiveness may be attributed (at least in part) to the upregulation of the ETA receptors in the lung tissue. In the present study, a dual ETA/ETB receptor blocker was applied to establish the involvement of ET-1 in hyperoxia-induced lung damage. The changes in the quantities of the specific receptors in this process requires the development of specific ET-1 receptor antibodies to visualize the altered distributions of the ETA and ETB receptors after hyperoxia. Although the application of specific receptor blockers in future studies may confirm the involvement of such a mechanism, the results of such experiments will furnish only limited information concerning the alterations in the quantities of the different receptor subtypes in response to hyperoxia.

Similarly to hyperoxia, TEZ also exerted dissociated effects on the airway and tissue compartments. It is noteworthy that in the naïve animals, the blocking of the ET-1 receptors with TEZ decreased the baseline Raw. This suggests the presence of a basal tone in the bronchial smooth muscle supplied by ET-1. Furthermore, TEZ reproduced the effects of hyperoxia on the airways (Fig. 1) in the naïve animals, confirming the involvement of ET-1 in the airway effects of hyperoxia. As regards the tissue mechanical parameters, the most noteworthy finding in the present study is the ability of TEZ to prevent the impairment induced in the lung parenchyma by hyperoxia. Numerous studies have demonstrated the involvement of ET-1 in various lung diseases that affect the airways [41] or the lung parenchyma [42]. The results of the present study provide evidence of the involvement of this peptide in hyperoxia-induced lung tissue damage. Moreover, ET-1 has been shown to have proinflammatory properties [43,44] and to promote edema development [43]. The current findings additionally demonstrate that blockade of the ETA and ETB receptors inhibits the proinflammatory activity of ET-1, which explains the efficiency of TEZ in protecting against hyperoxia-induced parenchymal mechanical impairments. The beneficial effects of TEZ against hyperoxia accord with the results of recent investigations revealing its preventive properties against the ET-1-induced increase in extravascular lung water [9], and the value of TEZ in the treatment of acute lung injury under experimental conditions in larger mammals [9,45] and in mice [46].

## Conclusion

In summary, the results of the present study yield evidence of the involvement of ET-1 in the lung function changes induced by hyperoxia. The highly dissociated effects of oxygen toxicity on the airway and tissue mechanics demonstrate the need for a separate assessment of the mechanical properties of these compartments in order to describe the alterations in the respiratory system accurately, and to design appropriate therepeutic strategies. The separate measurement of the airway and tissue responses revealed that, while hyperoxia induces a diminished reactivity of the airways to ET-1, the lung parenchyma exhibits a significantly enhanced constrictor response. Blockade of the ET-1 receptors by tezosentan prevented the lung tissue damage induced by hyperoxia. This demonstrates the key role of ET-1 in the lung damage evoked by oxygen toxicity, and suggests potential new perspectives for efficient prevention of the deleterious effects of hyperoxia on the lungs.

## Competing interests

The author(s) declare that they have no competing interests.

## Authors' contributions

WH conducted the design of the study and had a major role in drafting the manuscript. FP carried out the experiments on the major group of mice, performed the statistical analyses and participated in the manuscript writing. IRM participated in the study design and helped in processing the blood samples. YD helped in conducting the pretreatments and performing the experiments. JFD contributed in the design of the study. EL and GA carried out the experiments and data analyses on the adrenectomized animals. MB participated in the design of the study and interpretation of the experimental findings. CBA coordinated the various experimental approaches and contributed in their design. All authors read and approved the final manuscript.
